# 

**Published:** 1864

**Authors:** 


### CONTENTS.

### ORIGINAL CONTRIBUTIONS.

ART. XXXI. REPORT OF COMMITTEE ON PRACTICAL MEDICINE. By N. S. DAVIS, M.D., Prof. of Practical and Clinical Medicine,.......... 449
ART. XXXII. REPORT OF SURGICAL CASES. By GEO. K. AMMERMAN, M.D., Attending Surgeon to the Chicage City Hospital,.................. 458
ART. XXXIII. REPORT ON ORTHOPEDIC SURGERY. By DAVID PRINCE, M.D., Jacksonville, Ill. Presented to the Illinois State Medical Society. May, 1864, .................................................. 463

### SELECTIONS.

Animalculæ of Typhoid Fever, ................................................ 462
Death of a Medical Man in a Snow Storm, ................................... 462
A Plea for the Handmaiden. By Edward Parrish, .......................... 509

### BOOK NOTICES.

The Principles and Practice of Obstetrics. By Hugh L. Hodge, M.D., ... 513
Medical Diagnosis with Special References to Practical Medicine. By J. M. DaCosta, M.D., ............................................................ 513
A Manual of the Practice of Medicine. By Thomas Hawkes Tanner, M.D., F.L.S., &c., .............................................................. 513
The Medical Register of the City of New York, for the year 1864 By Guido Furman, M.D.,.......................................................... 513
Military, Medical, and Surgical Essays. By Wm. A. Hammond, M.D.,... 514
The Philosophy of Marriage. By Michael Ryan, M.D., ........................ 514
Alcohol and Tobacco. By James Miller, and John Lizars, .................... 514
Theory and Practice of the Movement Cure. By C. F. Taylor, M.D.,...... 514
A Treatise on the Chronic Inflammation and Displacement of the Unimpregnted Uterus. By Wm. H. Byford, A.M., M.D., ......................... 514
On Rheumatism, Gout, and Sciataca. By Henry Wm. Fuller, M.D., ...... 516

### EDITORIAL.

Explanation, ............................................................ 517
Suspension of Medical Journals, ................................................ 517
Medical Colleges in Chicago,...................................................... 517
The Case of Surgeon-General Hammond, ........................................ 518

